# Work-Life Stress during the Coronavirus Pandemic among Latina Farmworkers in a Rural California Region

**DOI:** 10.3390/ijerph19084928

**Published:** 2022-04-18

**Authors:** Annie J. Keeney, Amy Quandt, Daniela Flores, Luis Flores

**Affiliations:** 1 School of Social Work, San Diego State University, San Diego, CA 92182, USA; 2 Department of Geography, San Diego State University, San Diego, CA 92182, USA; aquandt@sdsu.edu; 3 Imperial Valley Equity and Justice Coalition, Calexico, CA 92231, USA; d309flores@gmail.com (D.F.); jr.luisf@gmail.com (L.F.J.)

**Keywords:** Latina, farmworkers, US–Mexico, stress, mental health, COVID-19, occupational stress

## Abstract

Objectives: To examine the type and severity of stressors experienced among Latina farmworkers during the COVID-19 pandemic. Methods: A survey containing the Migrant Farmworker Stress Inventory was administered to 77 female-identifying Latina farmworkers working in a US–Mexico border region. A sub-sample of five participants participated in key-informant interviews. Data collection occurred in Summer 2021. Results: Nearly 40% of Latina farmworkers reported high stress levels indicative of clinical mental health risks. Health and safety concerns and experienced stressors identified included visible substance abuse and poor bathroom conditions at the field site, language barriers, and balancing work and home life demands. Conclusions: Latina farmworkers have unique health and safety needs, and COVID-19 has contributed to the experienced stressors. Understanding the familial and working environment sources of stress specific to female agriculture workers is imperative to implementing culturally and gender-responsive strategies to better support the health and safety of farming populations in future pandemics.

## 1. Introduction

Work-related injuries are found to contribute to deaths from both opioids and suicides. For women, an occupational injury that led to at least a week off work tripled the combined risk of suicide and overdose [1]. Among foreign-born workers in the United States, Mexico-born workers in California have the highest rate of fatal occupational injuries, with death by suicide as the second leading cause of injury incurred [2]. Furthermore, death by suicide among foreign-born workers almost doubled from 2017 to 2018, in a non-pandemic year [2]. Increased mental health challenges and COVID-19 related stress associated with the loss of family members, reduction of income and work, and lack of personal protective equipment have been found among Hispanic/Latino, foreign-born farmworkers [3,4,5]. Studies have indicated that most people who die by suicide have a mental or emotional disorder, and depression and anxiety are associated with suicide attempts [6,7]. This is especially concerning given how historically, and now amid a global pandemic, Hispanic/Latino farmworkers are disproportionately burdened by worse health, mental health, and safety outcomes than other occupations in the United States.

While farmworkers are at increased risk of harm, of particular concern are the farmworkers in Imperial County, California. Located on the US–Mexico border, Imperial has the highest concentration of Hispanic/Latino populations in California and agriculture is the county’s largest industry, contributing $4.4 billion to the local economy annually [8]. Imperial County residents are five times more likely to work in agriculture when compared to other counties statewide and nationwide, and almost half of the farmworkers in the region are Mexico-born Latinas. Female headed households with children make up 62% of Imperial County households, which is almost double the state average [9]. Research has found that low-income Latina workers often have significant domestic responsibilities that result in increased levels of stress [10]. Furthermore, Latina agricultural workers are found to experience significantly higher stress and anxiety than non-agricultural female workers [11,12]. Given the large percentage of female headed households in Imperial, and one in six jobs are directly attributable to agriculture in the region, understanding how Latina farmworkers are experiencing stress during the COVID-19 pandemic in one of California county’s largest economic agricultural sector is imperative.

We aimed to measure the distinct stressors faced by Latina farmworkers working in a rural California region. Specifically, we sought to describe the type and severity of stressors experienced during the COVID-19 public health crisis and how/if those stressors relate to COVID-19 stressors. To our knowledge, this is the first study to quantify the personal and occupational stressors experienced among Latina California farmworkers in Imperial County.

## 2. Materials and Methods

During Summer 2021, we conducted an exploratory assessment, triangulating quantitative and qualitative data to assess the work and life stressors Latina farmworkers faced during the COVID-19 pandemic. The Migrant Farmworker Stress Inventory (MFWSI) was used to measure the stress experienced by our farmworker sample [13]. All items used a 5-point scale (“Have Not Experienced” to “Extremely Stressful”) and the total score was obtained by summing the scores for all the items. Possible scores range from 0–156 and individual scores of 80 or more indicate that the individual may have stress levels that pose significant mental health risks [13]. Cronbach’s analysis for the survey found excellent reliability (α = 0.994). We also added two items focused on sleep and air quality using the scale described above. The survey was administered via phone or in-person (*n* = 77); a sub-sample of survey respondents (*n* = 5) participated in key informant interviews. The key informant interviews focused on COVID-19 experiences (e.g., test and vaccine access, COVID-19 stressors) and perceived community support. Additional details on recruitment methods are available elsewhere [5].

We conducted analysis in SPSS (v. 27) (IBM SPSS Statistics, Chicago, IL, USA) and NVivo 12 (QSR International, Melbourne, Australia). Descriptive analyses were calculated for all items measured to characterize the distinct stressors faced by respondents. In analyzing the qualitative data, we identified concepts and themes based on the quantitative survey results (a priori), as well as those that emerged during analysis (a posteriori). We employed a cross-case analysis technique where we explored each interview individually and identified code words around concepts that emerged in the text. We examined the codes generated in the qualitative findings in relation to the survey findings to arrive at overarching themes and patterns [14,15].

## 3. Results

A total of 77 Latina farmworkers participated in the study. The age of respondents ranged from 18–65, with an average age of 45 years old. The vast majority were Mexico-born (78% vs. 22% US-born) and indicated their nighttime residence to be stateside (77% vs. 21% Mexico nighttime residence). Most respondents (82%) had a high school level education or less. Pre-existing health conditions were reported in a small portion of respondents: diabetes (13%), prediabetes (10%), high blood pressure (25%), and asthma (8%). Moreover, 36% of respondents reported testing positive for COVID-19 within the past year and more than 85% reported being fully vaccinated at the time of data collection.

### 3.1. Work Environment

The distinct stressors associated with the field site working environment were found to be significant stressors among the respondents in the survey and interviews. Drug use by others was the second highest perceived stressor among respondents (M = 2.60). Several respondents discussed witnessing firsthand substance abuse of co-workers either at the field or transportation site. Additionally, respondents also noted witnessing the effects of substance abuse on coworker’s working ability. For example, seeing a co-worker hungover at the field after a night of drinking. One respondent shared, "Drinking, smoking, staying up late, hanging out in the clubs around there, you know, that diminishes one physically, and that is reflected at work." Table 1 presents the top 10 stressors according to respondents based on survey items.

Additional elements such as weather, long work hours, and communicating in English were also among the top stressors experienced by respondents that were highlighted in the survey and interviews. Working in bad weather (M = 2.52) and working long hours (M = 2.52) were among the highest ranked stressors found among respondents in the survey instrument. When asked in the interview about things that cause stress at work, one respondent shared “Bad weather. When you arrive at a field and the field is very wet because it rained. Or the heat. Heat is another factor that you have to get used to. I mean, there is no option.” Another respondent replied “there are many work hours” when asked about feeling stressed at work.

The working environment associated with bathroom conditions was also found to be a prevalent stressor experienced by respondents. This stressor was reflected more in the interviews, however, was in the top third of overall stressors (M = 2.21) assessed in the survey instrument. “It isn’t just the weather that is (stressful)… because the other (stressor at work), are the bathrooms.” Several respondents also shared that daily cleaning/upkeep of the bathrooms in the field does not necessarily happen due to delays among the contracted cleaning companies. One respondent shared, “Look, the bathroom is one that you suffer because if they do not get to wash it, the bathrooms are despicable, they are horrible”. Bathroom conditions can cause a lot of additional stress among female workers. “One day, I went to a bathroom and came out vomiting because I inadvertently got nausea.”

### 3.2. Work–Home Demands

Overall, balancing the demands of work and home life was also a significant form of stress among respondents. Out of the top 10 stressors experienced based on the survey instrument, 3 of the 10 items were explicitly related to children or family concerns. These included worrying about who their children were spending time with (M = 2.50), children’s education (M = 2.48), and being away from family members (M = 2.46). These stressors of children well-being and being away from family were not further qualified in the interviews. Rather, we found much of the work–home (children and family) stressors were in relation to the COVID-19 pandemic. A predominant stressor among respondents was about bringing COVID-19 home to their family members. A respondent shared, “That was my fear, making sick those I had at home; that was stressful”. “It was stress in the form of not knowing if a person that you were talking to had COVID or not, had sick family members. Sometimes it was stressful to be taking care of you because logically, you don’t want to take COVID and bring it to your family.” Another respondent shared “Stress also hit me at the beginning of the pandemic… I was afraid of contagion or something, or me infecting my family”. Respondents also reflected on the stress experienced at the beginning of the pandemic, but with the availability of vaccines, this stress has subsided. “At first everything was fear, it was stress, but right now, look, because of the vaccines, my co-workers, most of them got vaccinated. And those that got sick, we are back, we came back and that’s it, stress went away little by little.”

## 4. Discussion

We found the working environment, the demands of balancing home and work, and stress associated with the COVID-19 pandemic concerning family as the most significant stressors experienced among Latina farmworkers in a rural California region.

Our findings support and extend the limited research on Latina farmworker mental health. To our knowledge, most research has been conducted in migrant and seasonal Latina farmworkers in North Carolina, Colorado, and Oregon and all before the onset of the COVID-19 pandemic [16,17,18,19]. In our study, we found nearly 40% of Latina farmworker respondents with stress scores indicative of significant mental health concerns. When comparing to other occupations, higher levels of depression and stress were found in Latina farmworkers, yet few were found to be at risk for alcohol dependence [19]. Our study’s finding that witnessing drug use among others was a significant source of stress is of particular interest. In 2019, it was estimated that one in five US residents reported being harmed by someone else’s drinking [20]. Harassment, aggression, property damage, and relationship issues have been identified as harms associated with secondhand alcohol use of others [20]. How this translates to farmworker populations especially regarding work relationships is unknown. The already inherent physical and emotional stressors associated with farm work and the increased behavioral health concerns related to the COVID-19 pandemic warrant an urgency to understand the effects of secondhand alcohol and drug use specific to farmworker populations and its impact on overall worker wellbeing.

Research has demonstrated that low-income Latina workers often have significant domestic responsibilities that result in increased stress levels [9]. We found that children and family concerns were a source of stress for respondents, with COVID-19 creating unique stressors. A main concern among respondents was exposing loved ones to COVID-19. We found that most respondents were fully vaccinated against COVID-19, and we speculate that the continued availability of the COVID-19 vaccine and boosters may help lessen this COVID-19 related stressor in the future given our findings.

We encourage agriculture employers and local farming associations to advocate for reducing stressors within their control and to take an active role in providing information on community-based resources. For example, increased attention to and maintenance of bathroom conditions at the field site could have an immediate positive impact for Latina farmworkers. Further, we suggest local child and family serving organizations, including public k-12 schools, to consider the unique ways farm work can contribute to family stress and parent mental health and, in particular, Latina farmworker head of households.

Although findings are specific to a unique geographical region, this is the first study to examine the occupational and personal stressors experienced among Latina farmworkers during the COVID-19 pandemic. Though the findings are from one case study, it provides a much-needed baseline on the distinct stressors and mental health risks experienced by California Latina farmworkers.

## 5. Conclusions

Given that one in six jobs in Imperial County are directly attributable to the agricultural industry [8] and 62% of households in Imperial are female headed [9], the mental health of the region’s female agricultural workforce is imperative. Understanding how to reduce stressors for Latina farmworkers in the working environment that will directly translate to homelife well-being is a pertinent issue for the entire community. As a first step, we recommend targeted behavioral health campaigns that provide information about community-based mental health resources at the field site. For example, bathrooms at the field site could have a hotline or a list of community resources posted that are available to help female agriculture workers navigate work and home stressors. Moreover, increased awareness or strategies on how to help cope with the secondhand effect of alcohol/drug use by others at the field site is necessary in improving agricultural health and safety efforts and reducing mental health risks for Latina farmworkers. Investing in Spanish-language access to these behavioral health resources at transportation or field sites is necessary to create a culture of health and safety within agricultural production that we feel could have a direct effect both for the individual worker and at the familial level.

## Figures and Tables

**Table 1:** Top Ten Work-Life Stressors Experienced by Latina Farmworkers.

Stressor	Mean	SD
1. Communicating in English	2.60	0.836
2. Drug use by others	2.57	0.759
3. Work long hours	2.52	0.766
Work in bad weather	2.52	0.877
4. Lack of sleep	2.51	0.775
5. Children spending time with	2.50	0.980
6. Children’s education	2.48	0.913
7. Unreliable transportation	2.46	0.969
Away from family members	2.46	0.820
Not feeling settled	2.46	0.788
8. No stores near by	2.45	1.005
9. Partner is absent from life	2.44	1.193
10. No medical care	2.40	0.782

## Data Availability

Restrictions apply to the availability of these data and are not publicly available due to IRB protocol and procedures.
